# Deciding Which Way to Go: How Do Insects Alter Movements to Negotiate Barriers?

**DOI:** 10.3389/fnins.2012.00097

**Published:** 2012-07-06

**Authors:** Roy E. Ritzmann, Cynthia M. Harley, Kathryn A. Daltorio, Brian R. Tietz, Alan J. Pollack, John A. Bender, Peiyuan Guo, Audra L. Horomanski, Nicholas D. Kathman, Claudia Nieuwoudt, Amy E. Brown, Roger D. Quinn

**Affiliations:** ^1^Department of Biology, Case Western Reserve UniversityCleveland, OH, USA; ^2^Division of Biology, California Institute of TechnologyPasadena, CA, USA; ^3^Mechanical and Aerospace Engineering, Case Western Reserve UniversityACleveland, OH, US

**Keywords:** barriers, central complex, electrolytic lesion, foraging in arena, insect brain, multi-channel recording, procaine injection, tethered walking

## Abstract

Animals must routinely deal with barriers as they move through their natural environment. These challenges require directed changes in leg movements and posture performed in the context of ever changing internal and external conditions. In particular, cockroaches use a combination of tactile and visual information to evaluate objects in their path in order to effectively guide their movements in complex terrain. When encountering a large block, the insect uses its antennae to evaluate the object’s height then rears upward accordingly before climbing. A shelf presents a choice between climbing and tunneling that depends on how the antennae strike the shelf; tapping from above yields climbing, while tapping from below causes tunneling. However, ambient light conditions detected by the ocelli can bias that decision. Similarly, in a T-maze turning is determined by antennal contact but influenced by visual cues. These multi-sensory behaviors led us to look at the central complex as a center for sensori-motor integration within the insect brain. Visual and antennal tactile cues are processed within the central complex and, in tethered preparations, several central complex units changed firing rates in tandem with or prior to altered step frequency or turning, while stimulation through the implanted electrodes evoked these same behavioral changes. To further test for a central complex role in these decisions, we examined behavioral effects of brain lesions. Electrolytic lesions in restricted regions of the central complex generated site specific behavioral deficits. Similar changes were also found in reversible effects of procaine injections in the brain. Finally, we are examining these kinds of decisions made in a large arena that more closely matches the conditions under which cockroaches forage. Overall, our studies suggest that CC circuits may indeed influence the descending commands associated with navigational decisions, thereby making them more context dependent.

## Introduction

As animals move through their environments, they must negotiate barriers that block their paths toward goals or away from threats. These challenges require changes in leg movements and posture as they execute appropriate maneuvers in the context of ever changing conditions. Thus, an animal must integrate both internal and external cues in order to appropriately alter local systems that re-direct movement. How do insects deal with these complex situations?

Contrary to the notion that insects are simple animals, they actually have at their disposal numerous sensory systems that monitor their own limb movements and their surroundings as well as a central nervous system that includes a sophisticated brain with several large and complex processing regions (Gupta, 1987; Strausfeld, 2012). Numerous studies indicate that insects use the information gained from visual (Pick and Strauss, 2005; Budick et al., 2007; Jeanson and Deneubourg, 2007; Duistermars et al., 2012), tactile (Blaesing and Cruse, 2004; Staudacher et al., 2005; Harley et al., 2009; Schutz and Dürr, 2011), auditory (Pollack and Pourde, 1982; Nolen and Hoy, 1986; Hedwig and Poulet, 2005; Poulet and Hedwig, 2005), and olfactory cues (Carde and Willis, 2008; Martin et al., 2011) to guide their movements in a context dependent fashion (Huston and Jayaraman, 2011). Moreover, aspects of physiological state may impact such decisions as certain goals may be more attractive to a hungry or thirsty insect than one that is satiated (Bell, 1990; Browne, 1993). In this review, we describe a top down strategy of behavioral and electrophysiological observations that begins to address this question. Our strategy relies on behavioral observations to document movements and generate neurobiological hypotheses. We then test those hypotheses using a range of electrophysiological recording methods. Finally, we return to behavioral studies in which various brain regions are lesioned or reversibly silenced and look for predictable behavioral deficits.

## Movement Around Barriers

As anyone who has had the misfortune of co-habiting with them knows all too well, cockroaches are very agile insects. In particular, they are adept at navigating all manner of barriers including blocks, shelves, holes, and walls in their attempts to reach goals or escape threats. Our first step toward understanding how they deal with these situations was to perform behavioral studies that quantified the cockroaches’ behavioral choices.

**Figure 1:**
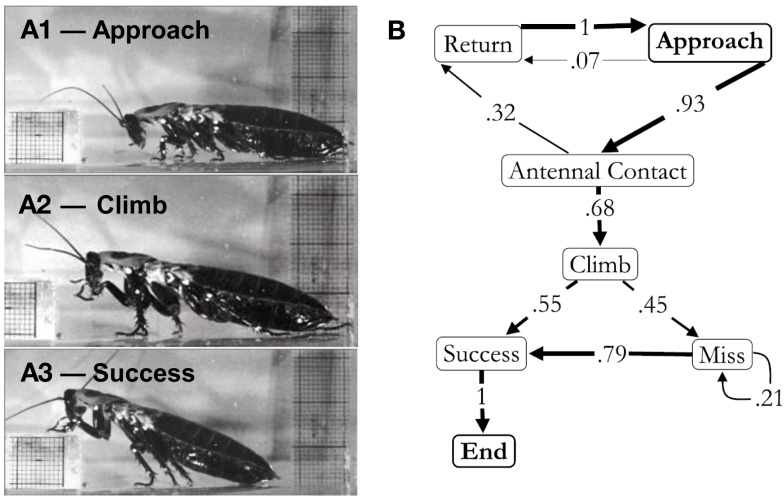
**(A)** Block climbing behavior: approaching the block **(1)**, swinging the leg to climb **(2)**, and climbing success **(3)**. **(B)** Ethogram of block climbing in the light. Arrows represent a direct transition from one behavior to the next. The number on the arrow and its thickness represent the frequency of that transition. This was calculated by dividing the number of times a specific transition was made by the total number of transitions exiting a specific element. All behavioral sequences began with the cockroach approaching the block (approach). It could then turn around and walk away from the obstacle (return) before or after antennal contact (antennal contact). The cockroaches would then enter a climbing sequence (climb), which could either be successful, with their foot reaching the top of the obstacle (success), or not be successful (miss). In the event that the cockroach missed, it would then produce another climbing motion, which again could either be successful or not. The end of the behavioral sequence occurred when the cockroach climbed the block. The beginning and end of the sequence must be “approach” and “end,” respectively. For this reason these elements are represented in bold. This sequence represents the responses of 58 individuals (one trial per individual). From Harley et al. (2009).

To accomplish this goal, we placed cockroaches in narrow tracks that contained individual barriers that had to be negotiated if the insect was to pass by. These could be blocks that had to be climbed over, shelves that could either be climbed over or tunneled under and bends that forced turning movements. Movement over blocks was examined in great detail (Harley et al., 2009). This behavior could be divided into a series of choices. For example, as the cockroach approached the block and touched the surface with its antenna, it could have stopped moving, turned around, or initiated a climb over the block (Figure 1). After looking at numerous trials, we assigned probabilities to each possible outcome. By following this process through the entire behavior until the cockroach was over the block, we generated ethograms that described the entire behavioral sequence in quantitative detail. The ethograms of these, and other behaviors, were critical to further studies. Without them, we would not have known whether the perceived changes that we recorded after a lesion or other procedure were part of the inherent variability of the behavior or a consequence of the manipulation. For block climbing, the ethograms showed that cockroaches approached the block and palpated it with their antennae. They then rotated their middle and front legs so that extension now pushed the body up and over the barrier (Watson et al., 2002). These climbing movements typically commenced well before any leg contacted the block and the degree to which the insect reared up was dictated by the height of the block. Thus, an intact cockroach moving at normal walking speed appeared to evaluate the barrier with sensors on its head and then acted accordingly rather than relying on reflexes generated by bumping into the object. The importance of the antennae in this behavior was clearly demonstrated by either shortening or removing them (Harley et al., 2009). Cockroaches with shortened antennae delayed climbing onset until the remaining antennal segments made contact, whereas individuals with ablated antennae reverted to simpler and less controlled strategies such as an elevator reflex that lifted the front legs ever higher each time they contacted the front of the object or even more simply by bulling forward until the head was forced over the object.

**Figure 2:**
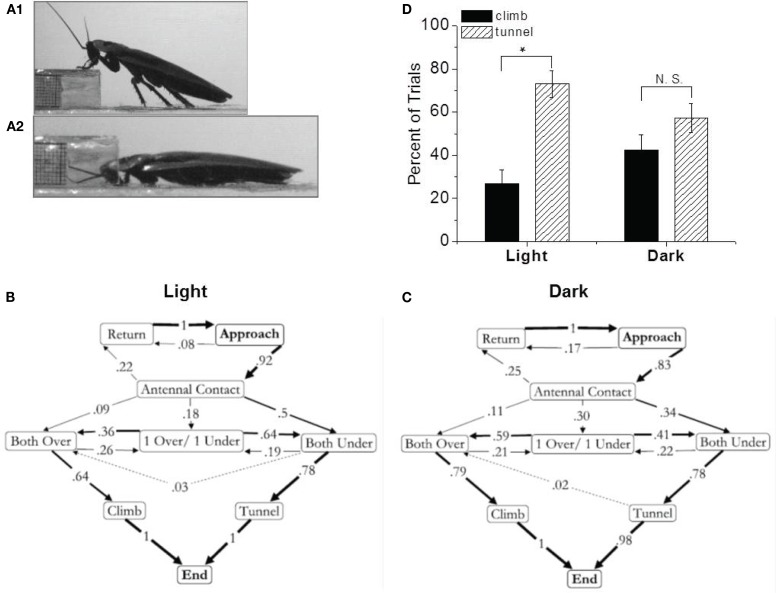
**Shelf climbing and tunneling is related to antennal contact**. **(A)** Pictures of climbing **(1)** and tunneling **(2)** behavior. Ethograms of shelf behavior in the light **(B)**, and dark **(C)**. Arrows represent a direct transition from one behavior to the next. The number on the arrow and its thickness represent the frequency of that transition. Dotted lines were used when two or fewer individuals preformed a specific transition. Antennal position relative to the shelf was determined as being both over the shelf (over/over), both under the shelf (under/under) or if one antenna contacted the top of the shelf and the other contacted the underside the pattern was recorded as (over/under). **(D)** Climbing and tunneling in insects presented with a shelf under different ambient lighting conditions. Naïve cockroaches were placed in the experimental arena with an obstacle they could climb over or tunnel under. The light condition represented 56 trials (14 climbs and 42 tunnels). The dark condition represents 61 trials (26 climbs, 35 tunnels). The error bars represent the ±standard deviation (±SD; calculated using methods for binomial data). In the light, the climbing and tunneling percentages are significantly different (*p* < 0.01, χ^2^ test). In the dark, this difference is not significant (*p* > 0.5, χ^2^ test). From Harley et al. (2009).

To create a more complex decision making paradigm, we replaced the block with a shelf (Harley et al., 2009). Now the cockroach had a choice. It could either climb over or tunnel under the object. Again, the resulting ethograms supported the central role of antennae in this decision making process (Figure 2). When the antennae contacted the shelf from above, the cockroach almost always climbed over it. When they contacted the shelf from below, the cockroach invariably tunneled under it. However, an interesting bias was detected in these data. About three quarters of the trials resulted in tunneling (Figure 2D). What could cause this bias? The cockroach’s inherent avoidance of light suggested an answer. Our initial observations were performed under bright lights. When we repeated them under low infrared lighting, the bias was no longer significant (Harley et al., 2009). Furthermore, under the original lighting conditions the bias could be eliminated by covering the ocelli but not by covering the larger compound eyes. Taken as a whole, these data clearly suggest that cockroaches use their antennae to negotiate objects in their path but in the context of ambient light, where bright lighting conditions bias the insect toward tunneling.

It is important to point out that while the ethogram studies that we describe above point to an important role for antennae in directing movement around, over or under barriers, they do not implicate specific sensory structures on the antenna. Antennae are very complex sense organs that contain numerous sensors including campaniform sensilla on the flagellum, hair plates at the base, and chordotonal organs as well as the Johnston’s organ (Staudacher et al., 2005). At this point, we cannot distinguish exactly which specific sensory receptors triggered transitions between individual stages of the behaviors described in these ethograms.

The behavioral importance of tactile cues detected by antennae is consistent with several other observations in both cockroach and stick insect. Okada and Toh (2000, 2006) have examined the role of antennal contact in the American cockroach as they navigate poles placed in their surroundings. Blinded cockroaches moving freely in an arena or tethered over a Styrofoam ball occasionally touch an object. They then approach it and often climb onto it (Okada and Toh, 2000). The scapal hair plates at the base of each antenna appear to be critical to this behavior, since shaving them increased the time to approach the object in unrestrained insects and impaired turning under tethered conditions. These researchers further described the active sensing movements of the antennae under tethered walking and showed that the position of a wooden dole relative to the body axis was correlated with the turn angle (Okada and Toh, 2006). Antennal sensing has also been shown to be important in gap crossing in stick insects (Blaesing and Cruse, 2004) in combination with tactile information from the front legs. More recently, the active sensing movements of stick insect antennae associated with climbing have been described in detail showing that leg movements are re-targeted as a result of tactual antennal information (Schutz and Dürr, 2011).

What about other sensory cues? In order to test competing directional signals, we set up another task. The insect was placed in a T-maze and we examined where it ended up (Kathman et al., 2011). In this experiment a transparent acrylic T-maze was constructed and placed over a mirror. The entry track was 12 cm long and connected to the middle of a cross track that was 20 cm long. The mirror allowed us to record the cockroach’s movements with a video camera at 60 fps. We then simply scored the number of times the cockroach ended up in the right or left arm of the T-maze then related those results to behavioral events such as the manner in which each antenna contacted the back wall. In some trials, computer generated moving black and white stripes were displayed on an LCD monitor placed behind the cross arm of the T-maze. Direction of the stripes was randomized. We then tested whether the visual pattern altered the number of times the cockroach followed antennal based turning rules.

In each trial, the cockroach walked down the entry corridor, touched the back wall with one antenna and 84% of the time moved to the opposite arm of the maze. That is, if the right antenna contacted the wall first, the cockroach ended up in the left arm and vice versa. The subject acted against this “touch-and-turn” rule in only 16% of trials, and the 84:16 ratio of turns away from the side of initial antennal contact is significantly different from chance (*p* < 0.01, Chi Square). With no other factors present, 50% of the time the cockroach ended up in the right arm and 50% in the left indicating that there was no inherent bias in the maze.

When we added the pattern of moving stripes to the back of the T-maze, the ratio of turning according to antennal contact was altered. If the stripes moved in the same direction as that dictated by the antenna touch-and-turn rule, there was little difference, the cockroach still predominantly turned away from the side where the antenna first contacted the back wall. If, however, the stripes moved in the opposite direction (e.g., stripes moved from right to left and the left antenna touched the back wall first) the tendency to turn with the antenna rule was reduced significantly (*p* < 0.05). Now only 60% of subjects turned with the antenna rule (down from 84%) and the incidence of turning against the touch-and-turn rule increased to 40% (up from 16%). This result suggests that an optomotor response generated by a pattern of moving stripes in the cockroach’s visual field can countermand the antennal touch-and-turn rule on some trials.

In light of the topic of this volume on invertebrate decision making, it is important to ask whether these actions are really decisions or are simply reflex driven behaviors. Most if not all of the behaviors we have discussed to this point could be explained by relatively simple reflexes. Even where two outcomes are possible (movement over or under a shelf), the “decision” is based primarily on the manner in which sensory structures, in this case antennae, contact the object. Greater insight into the distinction between reflex driven behavior and real decision making may come from examination of this entire volume. However, our sense is that unquestionable “decision” processes will be uncovered as we consider behaviors in more realistic situations, where the insect is free to choose among several possibilities and those choices are affected by environmental and internal conditions. We will discuss this notion further at the end of this review in the context of observations of cockroach behavior in a larger arena. We believe that the results of the more constrained behaviors, described above, will be components of those decisions, but this remains to be resolved through future experimentation.

## Sensory Integration in the Brain

These behavioral observations demonstrate that multiple sensory factors are used to guide the insect’s movement strategy (climb vs. tunnel) and direction (left vs. right turns). Clearly this requires a level of multi-sensory integration that must occur somewhere in the central nervous system. Given the use of head based sensors (antennae and eyes), the brain is a likely site for this process. Moreover, a considerable body of neurogenetic and electrophysiological data suggest that the central complex (CC) might play a role in this process (Huber, 1960; Strauss, 2002; Pick and Strauss, 2005; Ritzmann et al., 2008; Bender et al., 2010).

**Figure 3:**
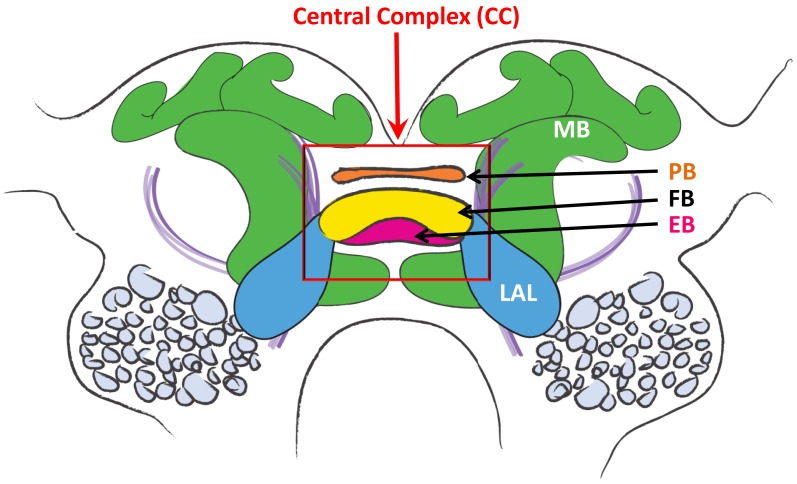
**Diagram of the cockroach brain**. Central Complex is within red rectangle. PB, protocerebral bridge; FB, fan-shaped body; EB, ellipsoid body; LAL, lateral accessory lobes; MB, mushroom body.

The CC is a set of interconnected neuropils situated in the midline region of the protocerebrum of virtually all insects (Figure 3; Homberg, 1987;Strausfeld, 1999, 2012; Wessnitzer and Webb, 2006). It includes the fan-shaped body (FB), ellipsoid body (EB), paired nodules and the protocerebral bridge (PB), which is dorsal and posterior to the FB, and links the two halves of the protocerebrum. Note: some laboratories use the notation of Central Body Upper (CBU) and Lower (CBL) divisions for FB and EB respectively. The FB and EB are believed to receive afferent fibers from multimodal sensory interneurons that in turn receive inputs from the various sensory neuropils of the brain. Fiber tracts link the EB and FB to the lateral accessory lobes (LAL), where contact is made with interneurons that descend to thoracic ganglia (Homberg, 1987, 2004) known to contain the local motor control circuits for walking and flying (Reichert and Rowell, 1985; Rowell, 1988; Burrows, 1996; Büschges and Gruhn, 2008; Büschges et al., 2008).

The CC is highly structured. In cockroach, the EB and FB each contain 16 columns, as does the PB (8 on each side of the midline). In histological sections, a very regular pattern of fibers can clearly be seen to connect the FB and PB of some species (Homberg, 1987; Strausfeld, 1999). Each pair of adjacent FB columns appears to receive inputs from two locations in the PB (Müller et al., 1997).

Based upon intracellular physiological and morphological data, Homberg and his colleagues developed a model that describes the flow of polarized light information from the FB or EB to the PB, and then out to the LAL and nodules of the locust (Heinze and Homberg, 2008, 2009; Heinze et al., 2009). Under this scheme, sensory information enters the FB and EB via tangential cells such as TL2 and TL3. Within the EB and FB are numerous polarized light sensitive columnar cells that form the columns and also project between neuropils (Vitzthum et al., 2002). In addition, tangential, amacrine, and pontine neurons cross columns horizontally within each neuropil (Müller et al., 1997; Heinze and Homberg, 2008). One horizontal class of polarized light neurons, called TB1’s, has dendrites that are arranged topographically within the PB columns according to the E-vectors to which they are sensitive, suggesting a topographic map of polarized light (Heinze and Homberg, 2007).

Considerable amounts of data also suggest that the CC plays an important role in supervising locomotion (Strausfeld, 1999). Earlier electrical stimulation studies implicated the CC in motor control. Huber (1960) examined movements of crickets walking on a ball and found that stimulation of the mushroom bodies via fine copper wires inhibited locomotion, while stimulation in the CC generated increased forward movement and turning. Our own studies, which will be described below, are consistent with these observations. Genetic manipulations also pointed to a role for the CC in locomotion control, in that several mutants that disrupt one or more CC neuropils have locomotor deficits. A *Drosophila* mutant called *no-bridge (nob)* has gaps in the PB and shows decreased frequency of walking bouts (Strauss et al., 1992; Strauss, 2002). Furthermore, when these flies do walk, steps are smaller and changes in step frequency do not occur as precisely as in wild type individuals. During turning, they may stumble rather than making smooth turns. Two additional mutant phenotypes that affect the PB in *Drosophila* initiate locomotion at normal rates, but for shorter durations (Martin et al., 1999). More recent neurogenetic studies indicate that a small subset of neurons in the EB, GABAergic ring neurons, are necessary for orientation memory in flies (Neuser et al., 2008) and that peptidergic neuromodulators can alter movements of flies within an arena (Kahsai et al., 2010).

With this background, we hypothesized that the antennal and visual cues that were critical to our barrier responses affect neural circuits within the cockroach CC that then alter movement through descending pathways. To test this hypothesis, we first had to establish that visual and mechanical sensory information reaches the CC. Because of the size of these structures (the combined EB and FB are ~400 μm × 200 μm in *Blaberus discoidalis*), we chose to address this very basic question with an extracellular multi-channel recording technique (McNaughton et al., 1983; Ritzmann et al., 2008). This technique has proven to be very useful, in that it allows us to record from numerous neurons simultaneously for long periods of time. Indeed, with some modification, we can even maintain our recordings while the insect is moving on a tether. However, as with any extracellular method, multi-channel recording does not provide the specific identity of the recorded neuron that intracellular techniques yield. We are limited to knowing simply where the extracellular electrodes were located at the time of the recording. Thus, a full assessment of the electrical properties of CC neurons will eventually require both intracellular and multi-channel electrical methods.

Since our initial goal in these studies was to establish whether visual and antennal information even reached the CC, we began with a restrained preparation. The insect was placed in a tube with its head stabilized by a wax covered plate. Each antenna was threaded through a hook that was connected to a servo motor. The servos were controlled by a custom computer program which, in turn, was controlled by user defined scripts. These scripts instructed the servos, for example, to first move one antenna medially for a predefined distance and velocity, then pause 5 s and return it laterally, then repeat that sequence 10 times. It then reiterated this process for the other antenna and finally repeated the entire routine one more time for a total of 20 movements of each antenna in each direction.

As with our behavioral studies, we did not attempt to determine exactly which sensory receptors on each antenna were stimulated. Since the entire antenna was pulled, the basal segments moved back and forth. This movement would certainly activate the hair plates that were shown to be important in orientation studies on the American cockroach (Okada and Toh, 2000). However, we cannot rule out that other sensory structures were also affected. In an attempt to isolate the stimulus onto the strain sensors of the flagellar segments, we placed a second hook just below the one that was attached to the servo. This bent the flagellum at one of three locations. Responses that were recorded at each site were similar to those recorded when the whole antenna was moved.

**Figure 4:**
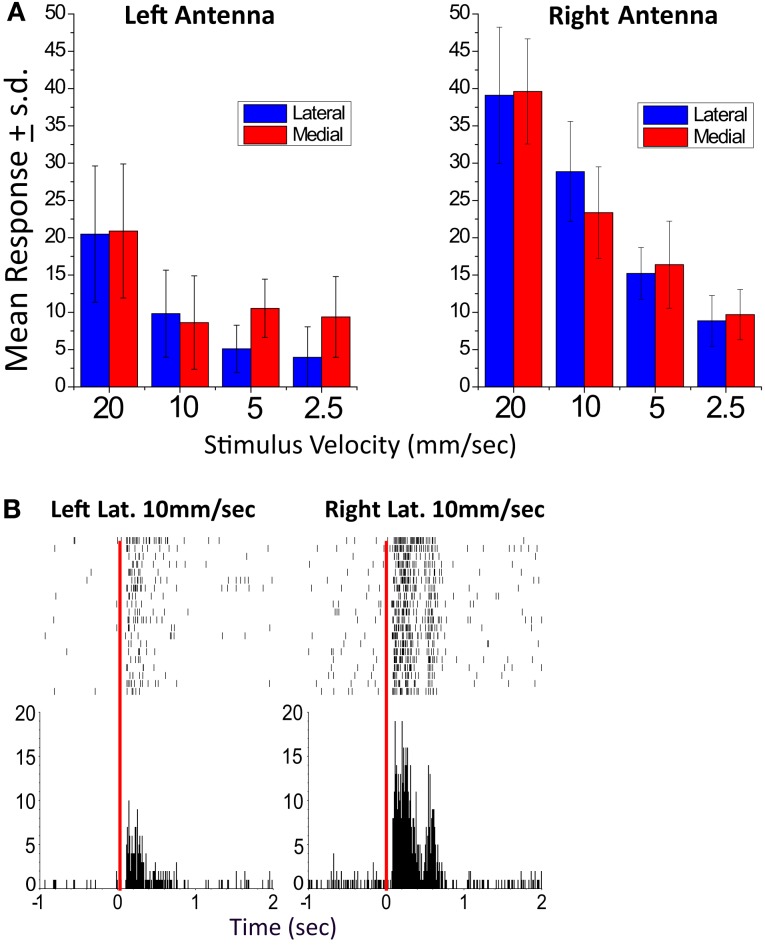
**A left-right biased unit recorded in the EB**. **(A)** Mean response graphs of lateral and medial stimulation of the left and right antenna. For this unit, all response values for stimulus paradigms of the left antenna were tested against the comparable paradigms of the right antenna (Student’s *t*-test). All were significantly greater for the right antennae (*p* < 0.01) except right and left medial 2.5 mm/s which was significant at *p* < 0.05. **(B)**. Sample left and right lateral raster display (10 mm/s stimulus velocity). In both records, the raster plots are of 20 responses of Left antenna moved laterally at 10 mm/s (left) and Right antenna moved laterally at 10 mm/s (right). In each case the top row is the first trial and the bottom the last. The time marks indicate the time that an action potential in this unit occurred. All records were lined up on the time at which the stimulation servo was activated (time 0). The histograms underneath the raster plots show the sum of all action potentials in 10 ms bins. From Ritzmann et al. (2008).

A time mark was saved each time the servo was commanded to move so that electrical records could be lined up accordingly (Figure 4). In these experiments, recordings were made by inserting a 16 channel silicon iridium probe into the brain in the region of the CC. The 16 channels on these probes were arranged in four tetrodes that sampled axons simultaneously. The shapes of the action potentials recorded at the four electrodes within a tetrode allowed us to separate responses from individual units off-line using cluster cutting software such as MClust. More details on this procedure can be found in the supplemental text of Bender et al. (2010). Typically we can record activity for 5–6 h from 5–6 units at each tetrode for a total of 20–24 units. After the experiment, the location of the probe was identified histologically.

The multi-channel recordings clearly demonstrated that both antennal and visual information do in fact reach the CC neuropils (Ritzmann et al., 2008). Most of the units recorded in either the FB or the EB responded to lateral movements of one or both antennae (Figure 4). Of these, the majority responded to either antenna with about one third showing a bias toward stronger activation from one or the other. We are confident that this bias was not the result of asymmetries in our stimulus system, because other units that were recorded simultaneously showed no bias or a bias to the other antenna. With these stimuli, we noted no spatial relationships between recording sites and response properties. The responses were typically velocity or acceleration sensitive. Velocity and acceleration sensitivity of CC units is important to the actual behavior, because when the cockroach touches objects with its antennae, it typically uses much lighter movements than those which we used in these initial experiments. Most antennal sensitive units were multi-sensory in that they also responded to changes in ambient light level. The majority of the visual responses were phasic, turning on with either increased or decreased light. Some were tonic units that showed much greater activity when the light was on than when it was off or vice versa.

More recently, we examined how neurons recorded in CC neuropils responded to moving stripe patterns (similar to those used in the T-maze experiments) that were projected onto a screen above the cockroach’s head (Kathman et al., 2011). These experiments utilized the same recording and analysis techniques as those that were employed for recording antennal responses in CC units. Several units recorded in the CC responded to moving stripes and a small subset of those units were sensitive to the direction of movement. Since many of these units were also sensitive to imposed antennal movement, we were concerned that the responses that we attributed to antennal movement might actually have been responding visually to movement of the antennal stimulation hook as it passed over the insect’s eyes. Indeed, for some units, responses persisted albeit at lower levels when the antenna was removed from the stimulation hook (Ritzmann et al., 2008). In these cases, the response was eliminated when ambient light was extinguished supporting the notion of a visual component. However, when antennal stimulation was conducted under these same dark conditions, the antennal response was typically unchanged. Thus, units within the CC appear to respond to *either* mechanical antennal stimulation or to visual cues with often little if any summation between them.

In the experiments described above, stimulation was imposed upon the antennae by the experimenter. In many systems sensory responses generated by the animal’s own active tactile movements produce very different responses than imposed stimuli (Prescott et al., 2011). We have, therefore, begun to examine responses in the CC to antennal stimulation produced by the cockroach’s own antennal movements toward an object (Guo et al., 2011). The recording techniques in these experiments employed two fine wire bundles that each formed a tetrode. These wire bundles are similar to those used in our locomotion studies described below and in Bender et al. (2010). Here, the cockroach is tethered in such a way as to permit normal walking and promote antennal searching movements toward a bar placed near its head. We used high-speed video to note when one of the insect’s antennae touched the bar. We then compared the responses of these self-generated contacts with those that occurred in the same unit when the antenna was tapped by the experimenter. Many units did not respond to self-generated antennal contact even though they may have responded to imposed stimulation; however others responded to both classes of stimuli. Where there was a response to self-generated contact, it typically contained fewer spikes than the responses to imposed stimulation. This difference is to be expected given the velocity dependence that is described above (Ritzmann et al., 2008). It may well be that the sparse nature of the self-imposed stimuli reflects a more realistic pattern that, over the entire CC population, provides more spatial information than that implied by the results from our stronger imposed trials.

## CC Influence on Locomotion

With the exception of the active sensing trials described above, our sensory studies were conducted in restrained preparations. In order to determine the relationship between CC activity and locomotion, we turned to a preparation in which the cockroach was tethered over a lightly oiled glass plate with a flexible plastic strip that allowed minimal up and down movement (Bender et al., 2010). Under these conditions, the cockroach walked in place with normal leg kinematics (Tryba and Ritzmann, 2000). High-speed video recording then allowed us to determine when the cockroach walked and, furthermore, to document changes in step frequency. Although the probes we used in the sensory studies were too delicate for these experiments, we could achieve similar multi-channel recordings from fine wire bundles implanted in the brain in a tetrode arrangement.

**Figure 5:**
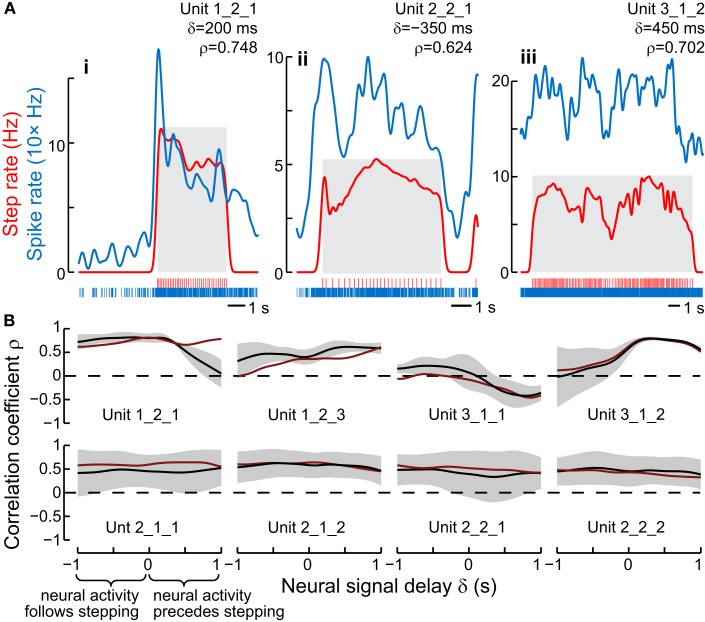
**Instantaneous stepping rate and neural firing rate were correlated in some units**. **(A)** The spike and step rasters were convolved with a Gaussian kernel (SD = 150 ms) to calculate instantaneous frequencies. The firing rate (blue) was shifted to the right by δ and cross-correlated with the step rate (red), leading to the listed maximum value of ρ (correlation coefficient) for each walking bout (gray boxes). Some walking bouts were elicited by a tap to the animal’s antenna, which evoked an additional response in some units **(i,ii)**. **(iii)** shows steps from an entire 16 s video in which the cockroach was walking before the recording started and continued after the camera’s memory was filled. **(B)** The correlation coefficient ρ changed as the spike rate curve was shifted relative to the step rate curve. The black line shows the mean and SD envelope for ρ at each value of δ. These 8 units were the only ones with a peak average absolute value of the correlation coefficient (|ρ|) of at least 0.4. The top row shows units with a peak |ρ| at δ > 0, meaning that changes in spike rate usually preceded changes in step rate. The units in the bottom row had flat curves with peaks at δ < 0. The red lines show the mean ρ calculated after removing the first 1 s of each walking bout to eliminate possible artifacts of antennal stimulation. From Bender et al. (2010).

We found several units that did change their response properties when the cockroach began to walk. Some of these units maintained their elevated firing level regardless of step frequency. However, other units in this class altered their firing rate along with step frequency (Bender et al., 2010). In order to examine this relationship more closely, we plotted two functions with time; the firing rates of each unit and the insect’s step frequency (Figure 5). As the step frequency changed spontaneously during the course of a recording session, the neural firing and step frequency curves paralleled each other remarkably well, maintaining high correlation coefficients (Figure 5A). Indeed, for a few of these units, the correlation coefficients increased when the firing frequency curves were shifted forward several hundred milliseconds relative to the step frequency curve (Figure 5B). This observation implied that the changes in firing frequency in these units occurred *prior* to changes in stepping frequency and could be part of the descending commands that act upon thoracic local control circuits to evoke increased step frequency. To establish a causal link, we then stimulated through the same electrodes that had been used previously to record neural activity (Bender et al., 2010). This stimulation typically increased the step frequency dramatically often with delays that were similar to the shift in spike frequency that produced the maximum correlation coefficients.

More recently we examined CC activity recorded in response to self-generated antennal stimulation (Guo et al., 2011). In this case, we used a different tether in which the cockroach walked on an air-suspended Styrofoam ball. By monitoring the movement of the ball, we could relate firing rate to forward walking movement and speed as well as to left and right turning. A bar was placed near the cockroach’s head where its antenna would occasionally tap it, causing the cockroach to turn. This paradigm simulates the touch-and-turn rule that we observed in the T-maze. The recording procedures were identical to those used in Bender et al. (2010).

Here we found CC activity that preceded turning or changes in forward walking with some consistent spatial relationships within the CC. Units recorded on the midline of the EB or FB tended to increase firing rate prior to forward walking or turning in either the right or left direction. However, units recorded in lateral regions of the EB and FB only increased firing rate prior to turns to one direction or to that direction and forward movement. Turns in the opposite direction were not coupled to significantly elevated activity in these units. As with walking speed, stimulation in these regions consistently evoked turning movements.

It should be noted that any changes in motor activity caused by the influence of CC neural circuits must act through the local circuitry that exists in the thoracic ganglia. This factor brings another level of complexity to this hierarchical arrangement of the central nervous system. Space does not allow us to discuss the properties of thoracic circuits other than to comment that in stick insect inter-joint reflexes may reverse when that insect walks backward (Akay et al., 2007) or turns (Hellekes et al., 2012). Similar reflex reversals can be generated by severing both of the neck connectives, thereby, eliminating communication with all brain regions (Mu and Ritzmann, 2008). The reader is referred to the following reviews on local circuits that control leg movements in insects for further information (Ritzmann and Büschges, 2007; Büschges and Gruhn, 2008; Büschges et al., 2008).

## Impact of CC Lesions on Behavior

Our recordings within the CC suggest that the tactile and visual information that we identified as important decision making factors in negotiating objects in the animal’s path are indeed processed within CC neuropils. Moreover, the walking preparations suggested that units recorded in these same neuropils can alter firing rate prior to changes in stepping frequency or directional movements, while activation of these units can evoke similar locomotory changes. With that information, we felt that it was necessary to return to our behavioral studies and ask whether manipulation of the CC neuropils could alter the various responses to barriers. Our results using electrolytic lesions and more recently reversible pharmacological silencing of neural activity complement and are consistent with *Drosophila* studies that use neurogenetic techniques to alter CC function (Pick and Strauss, 2005; Kahsai et al., 2010; Triphan et al., 2010).

We first examined the behavioral consequences of large mechanical lesions generated by inserting a foil lance into the CC or making sagittal cuts along the midline (Ridgel et al., 2007). These manipulations clearly showed major behavioral deficits associated with large scale damage within the CC. However, we felt that more discrete lesions were required to determine whether controls of individual behaviors are restricted to specific regions of the CC.

To accomplish this goal, we developed an electrolytic lesioning technique which could generate smaller lesions in discrete areas of the CC neuropils (Harley and Ritzmann, 2010). We examined the behavioral effect of a large number of these electrolytic lesions generated both within CC neuropils and elsewhere in the brain. Several behaviors were studied including climbing over blocks, climbing over or tunneling under a shelf, walking up a vertical wall then transitioning to a horizontal surface, and walking in a U-shaped track that required the animal to execute two turns typically generated by antennal contact. Each animal was tested before the lesion (pre-test) and after recovery from the surgery. Then the site of the lesion was identified histologically. The differences in each behavior were quantitatively scored and related to the lesion site.

**Figure 6:**
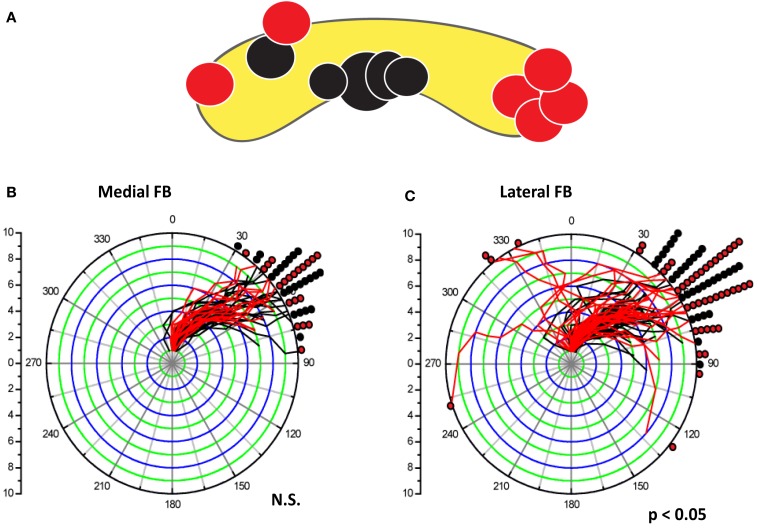
**Lateral lesions in the FB affected turning behavior in a U-shaped track, but medial lesions in the FB did not**. **(A)**. Location of lesions within the FB. Black dots showed no significant change in turning behavior between pre-tests and post-lesion trials. Red dots indicate lesions that produced 2 SD changes in the behavior between pre-test and post-lesion trials. **(B,C)** compare turning behavior of all pre- and post-lesion trials. For each trial, the change in turning angle of the cockroach body was measured relative to the position at antennal contact (plotted at the center of the polar plot) and at each subsequent step (annuli) made by the middle legs. Regardless of the direction of movement, curves that bend to the right indicate turns in the expected direction (away from the side-wall). Curves that move to the left would be cases where the insect turned into the wall. Any lines moving from the origin to the 0 angle would indicate no turn at all (no trials in these cases). The pre-lesion traces (black) were plotted under the post-lesion (red) curves. The final position data were divided into 10° bins. Black and red dots were used to mark the frequency of each final position. All pre-lesion trials turned in the expected direction. **(B)** All post-lesion curves for medial FB lesion were similar to pre-lesion curves. In contrast, post-lesion traces (red) in individuals with lesions in the lateral FB **(C)** showed increased variability after the lesion with several turns into the wall or turns that start in the wrong direction well before correcting later in to the trial. Data from Harley and Ritzmann (2010).

Most of the behavioral effects that we recorded in these experiments were restricted to the CC and for some behaviors, to specific regions of the CC. Lesions outside the CC tended to have little or no behavioral consequences in our tests. Controls in which the lesioning probe was inserted into the CC but current was not applied could generate some deficit but always at much lower levels than the electrolytic lesions. The spatial effects within the neuropil were demonstrated particularly well for turning behaviors associated with lesions within the FB (Harley and Ritzmann, 2010). Here 11 lesion sites were generated in separate animals. Seven of these sites were in lateral regions of the FB, while four were near the midline (Figure 6). The lateral lesions produced a significant increase in mistakes as the cockroaches navigated the U-shaped track. That is, when the cockroach encountered a section of wall that bent to the right, it should have turned in that direction to follow along the wall. However, a significant number of individuals with lesions in the lateral FB turned in the wrong direction (e.g., a bend to the right resulted in a turn to the left and into the wall). In contrast, the midline lesions produced no turning mistakes, but did result in errors in bilaterally symmetrical behaviors such as climbing over blocks or dealing with the shelf.

These electrolytic lesions demonstrated that CC neuropils play a role in controlling changes in locomotion. Although our controls supported the specificity of the lesions to CC neuropils, they came with the caveat that the probe may have damaged some tissue as it was inserted into the brain. We, therefore, sought to find a technique for *reversibly* silencing regions of the brain. We reasoned that reversible deficits could not have been caused by any permanent damage done during surgery or as probes were inserted into the brain.

**Figure 7:**
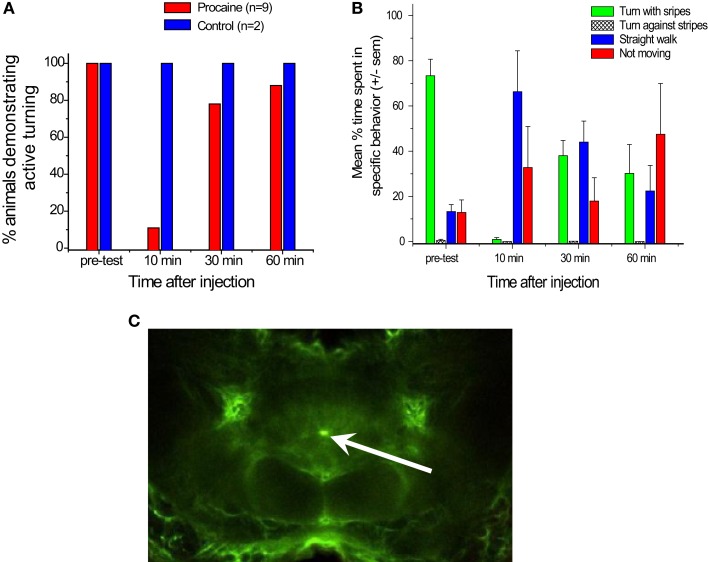
**Injection of procaine into the central complex alters turning behaviors**. **(A)**. Nine cockroaches were tested for active turning movements in the T-maze before and after injection of 20% procaine (red bars) or saline (blue bars). “Active turns” were defined as cases where the cockroach rotated its body after touching the wall with its antenna but before its head hit the wall. These movements contrasted with passive actions, where the head hit the wall and moved along the wall’s surface as the cockroach appeared to try to walk forward. Some passive turning movements occurred while these cockroaches continued to force themselves forward, but were easily distinguished from active movements. Note that prior to injection, all trials resulted in active turning, but 10 min post-injection the percentage of active turning was greatly reduced. Recovery started at 30 min and was nearly complete at 60 min. Two controls in which saline was injected into the central complex in the same way showed no changes after the injections. **(B)**. Responses of five cockroaches walking on an air-suspended ball to a moving stripe pattern in their visual field. Bars indicate average percentage of time spent turning with the stripes (green), turning against the stripes (hatched), walking straight ahead (blue) or not moving at all (red). Note that in the pre-test most trials showed the cockroach walking with the moving stripes, while 10 min after injection of 20% procaine few trials followed the stripes and many more showed straight or no walking. At 30 and 60 min, there was some recovery, but not completely back to normal. **(C)**. Confocal image of a brain injected with procaine mixed with fluorescein dextran for six pulses at 40 ms and 30 ψ. The bright fluorescein dot at the center of the central complex between the fan-shaped body and ellipsoid body shows the injection site.

We turned to a procaine injection technique that had been used by other laboratories to generate reversible deficits in other regions of the brain (Devaud et al., 2007; Gal and Libersat, 2010). Procaine is a voltage gated Na^+^ and K^+^channel blocker that reversibly silences action potentials. We pressure injected 20%procaine mixed with fluorescein dextran into the CC neuropils while recording with our multi-channel electrodes and found that it silenced all units in the immediate region of the injection site for about 30 min (Pollack et al., 2011). The fluorescein allowed us to identify the injection site histologically (Figure 7C). In some cases the effect was restricted to units recorded at one pair of tetrodes. In locust, glial sheaths surround the CC and also project into CC neuropils (Boyan et al., 2011, 2012). We observed similar structures in cockroach which could have blocked migration of the drug to the other tetrodes After 30 min, activity returned to what appeared to be normal levels.

The behavioral consequences of procaine injection in the CC were profound (Pollack et al., 2011). For behavioral studies, we injected 20% procaine in saline with single 120–150 ms pressure pulses or multiple pulses of 30–40 ms. Within 10 min of the injection, the cockroach’s walking and navigational behaviors were dramatically reduced. In the T-maze, insects failed to turn when their antennae contacted the wall (Figure 7A). Rather, they tended to walk along the wall with their heads pressed against it, something that normal intact cockroaches rarely, if ever, do. At 30 min after injection, the animals showed some recovery and by 60 min, they were close to normal behavior. On the ball tether, procaine injected cockroaches either failed to walk at all or walked straight ahead even as moving stripe patterns that routinely generate continuous turning movements were projected onto a screen in their visual field (Figure 7B). Again, within an hour, the cockroaches had begun to show signs of recovery. In both of these paradigms, injection of saline in the same manner had no effects.

## What is the CC’s Role in Navigating Complex Terrain?

Our data point to a role for CC neuropils in decisions made by cockroaches as they navigate complex terrain. Our behavioral data demonstrate that mechanical antennal information along with visual cues guide these decisions as the cockroach walks through a track and encounters a barrier. These types of sensory cues are processed in CC neuropils where units are found whose activity appears to influence locomotory changes. Moreover, both permanent and reversible lesions in the CC have dramatically altered these same behaviors.

These results strongly suggest that activity descending from the CC interacts with local control circuits in the thoracic ganglia to re-direct leg movements. They are consistent with neurogenetic reports regarding the role of CC neuropils in *Drosophila* locomotion (Strauss, 2002; Pick and Strauss, 2005). But the question still remains, what is the precise role of the CC in this command structure? Certainly, simple orientation movements such as escape or wall-following could take place with much simpler reflex circuits. Most of the circuitry that controls escape turns in the American cockroach, *Periplaneta americana*, resides in the thoracic and abdominal ganglia (Ritzmann and Pollack, 1988, 1990; Ritzmann, 1993; Ritzmann and Eaton, 1997). Although activity descending from the head ganglia does affect escape responses (Fouad et al., 1994; Schaefer and Ritzmann, 2001; Libersat et al., 2009), the turn direction arises primarily from the direct influence of directionally sensitive ventral giant interneurons on thoracic ganglion circuits (Levi and Camhi, 2000a,b). Antennal responses to tactile stimulation activate descending interneurons that evoke similar escape turns (Burdohan and Comer, 1996; Comer et al., 2003; Ye et al., 2003), but again, these interneurons appear to act through fairly simple direct activation of the thoracic interneurons that generate the escape turns. Similar antennal circuits could be envisioned to control rapid turns made by cockroaches as they run while maintaining contact with a wall (Camhi and Johnson, 1999; Cowan et al., 2006). If these rapid turning movements could be controlled by simple almost reflexive connections from sensors to local motor circuits that control legs, why does the cockroach, or any other insect, need something as large and intricately structured as the CC?

The answer to this question could reside in the multi-sensory nature of the behaviors that are disrupted by CC manipulation (Harley et al., 2009; Harley and Ritzmann, 2010). But here again, the escape system also is multi-sensory. The cockroach can escape equally well from tactile stimuli as from wind directed at the cerci (Comer et al., 1994; Schaefer et al., 1994; Stierle et al., 1994), and again this capability stems from convergence of tactile interneurons with the same thoracic interneurons that receive input from the wind sensitive ventral giant interneurons (Pollack et al., 1995).

**Figure 8:**
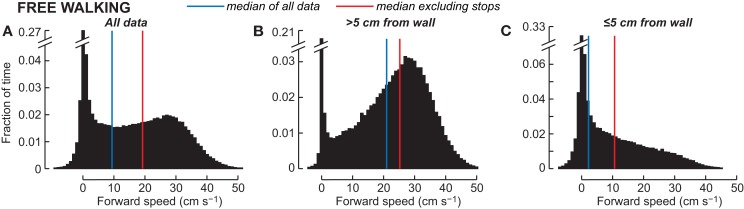
**Forward walking speed in the empty arena**. **(A)** Pooled histogram from 44 animals, ~1 min of walking each at 20 samples s^-1^. **(B)** Speeds when the animal was more than 5 cm from a wall, approximately the radius of antennal contact (*n* = 31,200 samples). **(C)** Speeds when the animal was less than 5 cm from the nearest wall [**(A,B)**; *n* = 28,464 samples]. The blue vertical lines indicate the median walking speed in each histogram; the red lines show the median speed when data with an absolute value less than 1 cm/s are excluded. From Bender et al. (2011).

The difference between the behaviors that are associated with direct sensori-motor connections in the thoracic ganglia and the kinds of behaviors that the CC is involved in may be temporal. Control of escape movements or even rapid wall-following must occur in the millisecond range. But foraging decisions can occur over much longer time frames. A study of walking speeds in a large arena demonstrated that the cockroach spends most of its time walking relatively slowly around in its environment exploring with its antennae while taking in tactile, visual, and olfactory cues, and then moving accordingly (Bender et al., 2011). Indeed, walking in that arena clustered around two speeds (Figure 8); a slow ambling gait (<10 cm/s) and a less common faster trotting gait (~30 cm/s). Even these trotting gaits occurred at speeds well below the median escape velocities recorded in the open arena (41.1 cm/s). A breakdown of the time that the cockroaches spent moving at these different speeds showed that when they were near the wall of the arena (their preferred location), most of their movements were in the slowest range. When they were in the middle of the arena, they sometimes changed to the faster trotting gait, possibly to get back near a wall. Thus, the cockroach spends most of its time moving slowly through its environment examining objects and reacting accordingly. The rapid movements associated with escape are, in fact, rare occurrences that happen only in the face of an imminent threat. So, while there is no question that these rapid behaviors are critical to the cockroach’s survival and are very useful experimentally in working out reduced neural circuits and biomechanical properties (Jindrich and Full, 2002; Koditschek et al., 2004), we need to appreciate the relatively small part they play in the insect’s behavioral life. Indeed, even in escape responses, it may only be the initiation of movement that is evoked by these relatively simple connections. More recent analysis of cockroach escape demonstrates that after the initial turn, movements become unpredictable (Domenici et al., 2008). Moreover, in *Drosophila* leaning movements directed away from a visual threat before the escape is triggered appear to incorporate some degree of motor planning that may involve the CC (Card and Dickinson, 2008).

When we begin to consider the slower and more complex decisions that occur during normal foraging, new hypotheses begin to arise for the role of the CC and other brain structures. In contrast to relatively simple escape circuits and behaviors, the CC seems to be positioned to take in massive amounts of information about an individual’s surroundings and possibly also its internal state, then use this information to influence movement so as to match locomotory changes to current environmental and internal context. It is possible that CC circuits do not, on their own, evoke any changes in movement. Cockroaches with lesions within the lateral regions of FB, that were associated with most of the turning errors we scored, still showed many correct turns (Harley and Ritzmann, 2010). Thus, even these lesions did not completely disrupt the insect’s ability to turn properly. Moreover, the neurons that exit the CC do not typically connect directly to the thoracic ganglion but rather project to areas such as the LAL where they encounter more direct descending neurons (Heinze and Homberg, 2008). This pattern suggests that CC circuits *influence* descending commands rather than evoke them directly. This model is consistent with Strausfeld’s (2012) notion that the CC serves as a “brain within the brain” to integrate information about what is currently occurring and then fine tune behavior to current conditions. He points to observations in the wasp sting story as support. The jewel wasp, *Ampulex compressa*, stings an adult cockroach first in the prothoracic ganglion and then in the brain (Fouad et al., 1994). The first sting briefly paralyzes the cockroach, but the more long-term second sting prevents volitional or escape movement rendering the cockroach a virtual automaton. In this state, the wasp will pull on the cockroach’s antennae and the cockroach will then follow the wasp into its nest where it will be entombed with the wasp’s egg to serve as food for the new, developing wasp. Studies using radioactive tracers demonstrate that this second sting does in fact inject venom near the CC and mushroom bodies (Haspel et al., 2003).

If the CC plays a role in matching movement to external and internal states, one would expect that modulatory substances would influence the states of CC circuits and that appears to be the case. Numerous neuromodulators and their receptors have been found within the CC’s of various insects (Homberg, 1991; Nässel and Homberg, 2006). One study used genetic tools to manipulate the transmission of *Drosophila* tachykinin from interneurons that innervate the CC (Kahsai et al., 2010). The affected flies significantly reduced their tendency to avoid the central zone of a test arena. The authors concluded that “…peptidergic pathways in the CC have specific roles in the fine tuning of locomotor activity in adult *Drosophila*.” Consistent with these neuromodulatory effects, we noted that response patterns of units recorded in the CC during our multi-channel studies often varied dramatically over the course of a 5 h recording session (Ritzmann et al., 2010). These changes were typically not consistent from one unit to another even when they were recorded simultaneously. Rather some units increased sensitivity to antennal stimulation while others decreased or stayed the same. This finding could suggest that the CC moves through various different states in response to physiological transients (e.g., hunger, thirst, fatigue, aggression or attention) which could be associated with release of neuromodulators or hormones. Similar state changes are well-known in more thoroughly studied structures such as the stomatogastric ganglion of crustacea (Harris-Warrick et al., 1992; Meyrand et al., 1994). Thus, it is possible that circuits within the CC monitor sensory cues surrounding the insect as well as internal physiological state and then modify descending commands to match decisions to current context.

If the principal role of the CC is to fine tune foraging movements in complex environments, we may have to move to more complex behavioral paradigms to understand it. The behaviors that we have examined so far required the cockroach to make only limited choices. We designed those experiments so that we could deal with manageable variables in our behavior and lesion studies, however, they may not have taxed the cockroach enough to reveal the CC’s primary function.

*Movement in an Arena:* For this reason, we began to observe cockroach behavior in a more enriched situation. We allowed them to seek a darkened shelter in a well-lit 90 cm × 90 cm arena. Most cockroaches (including *Blaberus*) are extremely photonegative (Meyer et al., 1981; Okada and Toh, 1998; Canonge et al., 2009), and thus the dark shelter attracted the animals in about half the time than would be expected based on control trials in an empty (no shelter) arena (Daltorio et al., 2012). However, as noted above, they are also known for thigmotaxis (Okada and Toh, 2006; Halloy et al., 2007; Nishiyama et al., 2007), spending much of their time along the walls of such arenas while relying on antennal contact to maintain a constant wall proximity (Camhi and Johnson, 1999; Cowan et al., 2006). Thigmotaxis would dictate staying on the wall while photonegative tendencies would have the cockroach leave the wall and move directly to the shelter. How are these seemingly conflicting behaviors resolved? Is an environmental map required? Does the cockroach plan the best paths? Alternatively, can the total behavior occur as a result of the insect continually updating the direction of its movements based upon some relatively simple rules?

**Figure 9:**
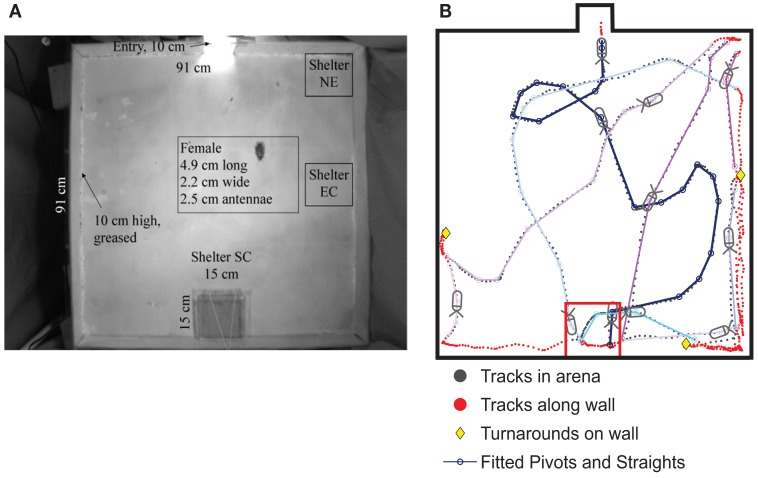
**(A)** Cockroaches were released at an entry point (top) of an enclosed well-lit arena and permitted to seek darkened shelters in three locations: 33 trials with the shelter in the center of the midback wall (referred to as SC), 17 trials with the shelter in corners closest to the top (NE), 40 trials with the shelter along the side-wall (EC), and 44 trials with no shelter, as a control. **(B)**. The cockroach’s path was tracked using the CTrax automated tracking system. A sample track shows the cockroach arriving at the SC shelter after a series of three turns, which we fit to pivots and straights. It then left the shelter and followed several additional (colored) tracks out to the arena and back to the shelter. Those later tracks spent more time along the walls, which is typical of cockroach behavior in the arena. From Daltorio et al. (2012).

When the cockroaches encountered a wall in the arena, they generally followed it according to the touch-and-turn rule observed in the U-track and T-maze. Occasionally, they changed direction along the wall or departed it to explore the interior of the arena (Figure 9A), however, these actions did not appear to be specifically directed toward the shelter as might be expected if they were using an internal map or some other long-term strategy. Rather, they seemed to be continuously updating their situation relative to the competing goals of wall-following and shelter-seeking.

**Figure 10:**
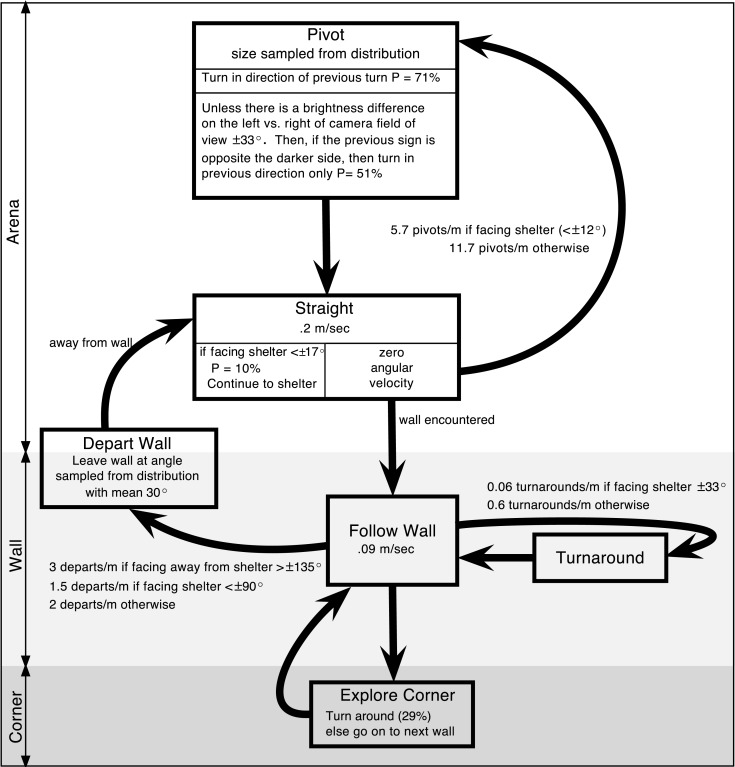
**Proposed state-based diagram of RAMBLER, randomized algorithm mimicking biased lone exploration in roaches**. There are states for along the wall, in the corner, and in the arena. “Follow Wall” continues until a turnaround or departure is randomly selected at shelter-orientation dependent rates or until a corner is encountered. “Straight” continues until a wall is encountered or a pivot is randomly selected. Based upon figure in (Daltorio et al., 2012).

To test the hypothesis that this behavior did not rely on an internal map or long-term strategy, we fit the insects’ continuous turning movements to a biased persistent random walk (Figure 9B). The insect was modeled as having a finite group of states: “pivot” or “straight” in the center of the arena and “follow wall,” “turnaround,” or “depart wall” when along the wall. Each state had an associated behavior: walk in a line for “straight,” turn in place for “pivot,” and maintain constant wall distance for “follow wall,” etc. Transitions from one state to another occurred stochastically based on state transition rates, which were extracted from the cockroach data. For example, to quantify the connection between the “follow wall” state and the “depart wall” state, we measured the wall departure rate of two departs per meter by counting the number of times animals left the wall and dividing by their total path length along the wall. Since we measured the speed of the animal along the wall to be ~0.09 m/s, over a given time period of say, 05 s, there is a 2_*_0.09_*_0.05 = 0.9% chance of a wall departure. To understand how vision affects wall-following, we parsed the data in MATLAB by the angle to the dark shelter for three different shelter locations (Figure 9A) and compared the results to empty arena data. We found that the animal was only more likely to leave the wall when the shelter was behind the animal (at egocentric angles of 144–180°). Similarly, the fitted insect tracks provided us with transition rates between the other states. The manner in which these parameters vary with the perception of the shelter defines our model, RAMBLER (Randomized Algorithm Mimicking Biased Lone Exploration in Roaches; Figure 10). Specifically, we found the following trends to be statistically significant (90% bootstrap confidence intervals not overlapping):

1. Depart wall more frequently if the shelter is behind the insect
2. Change direction on the wall less when facing a darkened shelter
3. Turn less when facing a darkened shelter (e.g., two long straight periods in initial track of Figure 9B)
4. Turn more to counter previous turn if the shelter is detected on the opposite side (e.g., last turn in the first track of Figure 9B)

When we simulated the entire model with and without a shelter, we found that these trends were sufficient to capture much of the shelter-seeking bias. Figure 10 is a diagram that depicts the state-based algorithm that, in simulation, captured the behavior of the cockroach in the shelter-seeking task (Daltorio et al., 2012). The success of this model supports the hypothesis that insect decision making (at least in this context) is based on current perception rather than a multistep map-based plan. It is interesting to note that this model required little memory and we found no evidence that the cockroach was planning its route.

The level of complexity of the RAMBLER algorithm is an indication of the multi-sensory dependencies we expect to find in the insect’s brain. In our trials, the animal neither blindly followed the wall nor perfectly tracked the goal. When wall-following, the animal was more likely to depart when the shelter was behind it. This shows that even while relying on antennal feedback to maintain the proper wall-following distance, the insect evaluated the changing visual response to decide when to leave the wall. When the antennae were not in contact with the wall, the turns the insect made may at first glance appear random. However, our analysis showed that when the shelter was in front of the cockroach, it turned away less frequently, and was more likely to correct turns away from the shelter. The presence of a visual goal seemed to modify the normal turning and wall-following behaviors to correct for undesirable changes in the animal’s perception. Eventually, the subjects almost always reached the shelter, but they did not always stay there (Figure 9B). Indeed, they often left and returned several times. This observation in itself suggests that the cockroach was not dominated by a single-minded goal to reach the darkened shelter, but rather continuously considered several factors as it moved.

For this task, a more reflex driven algorithm that directed the cockroach toward the shelter once it saw it might actually be more efficient. However, as more competing goals (food plus shelter plus mates) are added into a more complex environment with barriers to those goals, the more directed model may not be as robust as the process that the cockroach appeared to utilize. These more complex situations would probably be closer to what the insect faces in nature.

As we begin to understand this relatively simple environment, we will add more features to try to capture the decision making that the cockroach uses as it forages in natural environments. We also must consider social interactions that occur when multiple cockroaches are present (Halloy et al., 2007; Jeanson and Deneubourg, 2007). Finally, we hope to apply the neurobiological recording, lesion, and procaine techniques to these studies. Meanwhile, we have implemented RAMBLER on a small, wheeled robot to navigate unknown environments with visual goals and tactile barriers, which it does in a remarkably insect-like fashion (Daltorio et al., 2012).

## Conclusion

Our research on decision making in cockroach locomotion has followed a very multi-level approach. We are convinced that a thorough understanding of how insects deal with the challenges of moving through natural environments requires all of the experimental paradigms that we and others have at our disposal. We start with behavior that leads to neurobiological hypotheses that are tested with electrophysiological techniques. The results of these experiments suggest that specific regions of the central nervous system play important roles in controlling these behaviors, leading to lesion studies that examine behavioral deficits. While relatively simple behavioral choices play an important role in defining the decision processes, we feel very strongly that we must also examine more realistic situations that truly capture the parameters of foraging behavior. Other studies such as the neurogenetic observations in *Drosophila* greatly influence our thinking. We are a long way from understanding the exact role of the CC in this process, but we believe that we are on the right track.

## Conflict of Interest Statement

The authors declare that the research was conducted in the absence of any commercial or financial relationships that could be construed as a potential conflict of interest.
